# Association Between Body Mass Index and Oral Health Status Among Children: A Cross‐Sectional Study Contributing to Policy‐Driven Improvements in Children’s Oral Health

**DOI:** 10.1155/ijod/2332068

**Published:** 2026-04-10

**Authors:** M. Alfiya, Rekha P. Shenoy, Imran Pasha M., Junaid, Supriya Amanna

**Affiliations:** ^1^ Department of Public Health Dentistry, Yenepoya Dental College, Yenepoya (Deemed to Be University), Mangaluru, Karnataka, India, yenepoya.edu.in

**Keywords:** body mass index, child, dental caries, nutritional status, oral health

## Abstract

**Background:**

Dental caries, periodontal disease, and malnutrition adversely affect children’s growth and development and are recognized as significant public health concerns. The link between child nutrition and oral health is crucial, as they share multiple risk factors. Assessment of nutritional status using standardized growth references such as the Indian Academy of Pediatrics (IAP) growth charts is essential, as variations in body mass index (BMI) have been associated with differences in oral health outcomes among children.

**Objectives:**

To evaluate the association between BMI and oral health status in children aged 12–15 years attending a tertiary care hospital in Mangaluru, Karnataka and to highlight implications for integrating nutritional screening into oral health prevention programs.

**Methods:**

A cross‐sectional study was carried out among 164 children aged 12–15 years attending a tertiary care hospital in Mangaluru, Karnataka. Children who provided assent and whose parents gave written informed consent were included in the study. Anthropometric data, including height and body weight, were recorded and BMI was calculated. Oral status was evaluated using the World Health Organization (WHO) Oral Health Assessment Form for Children, 2013. Statistical analyses were performed using SPSS version 26.

**Results:**

Among the 164 participants, overweight/obese children had significantly higher decayed, missing, and filled teeth (DMFT) index and increased gingival inflammation compared to normal and underweight groups (*p*  < 0.01). A positive correlation was observed between BMI and DMFT scores (*r* = 0.377), as well as between BMI and periodontal status (*r* = 0.34). Urgency of intervention was also significantly associated with BMI.

**Conclusion:**

The findings suggest that nutritional status may be an important indicator of oral health among children aged 12–15 years. Integrating BMI assessment into routine dental and school health programs may support early identification of at‐risk children and facilitate comprehensive preventive strategies.

## 1. Introduction

Oral health is a key indicator of overall health and quality of life, encompassing conditions like dental caries, periodontal disease, tooth loss, oral cancer, oro‐dental trauma, and congenital anomalies such as cleft lip and palate. Due to factors like urbanization, poor access to dental care, insufficient fluoride exposure, and the pervasive promotion of alcohol, tobacco, and sugary foods—all of which are linked to poor oral and overall health—the prevalence of oral illnesses is rising in many low‐ and middle‐income nations [1]. A key factor in enhancing general health outcomes is early diagnosis of oral disorders. In addition, a comprehensive oral examination can identify systemic problems such immunological disorders, infections, trauma, and nutritional deficits [2].

Dental caries is caused by microbial demineralization of tooth structures and, if left untreated, may lead to pain, discomfort, and eventual tooth loss, particularly in children [3, 4]. Dental caries in permanent teeth is the most common oral disease globally and affects ~3.5 billion people. This includes over 530 million children with caries in permanent teeth [1]. The prevalence of dental caries varies from 49% to 83% across countries as reported in previous literature [5]. The burden of untreated dental caries continues to be substantial, affecting both primary and permanent dentition, particularly in children and adolescents [6]. Globally 60%–90% of schoolchildren and nearly all adults are affected, with higher prevalence reported in Asia and Latin America. In India, the National Oral Health Survey (2002) reported dental caries in 52.5% of 12‐year‐old children [7, 8]. Periodontal disease affects the supporting tissues of the teeth and manifests as gingival inflammation accompanied by bleeding and swelling. In advanced stages, it results in loss of supporting bone and tooth mobility. Poor oral hygiene is a key factor and periodontal conditions contribute substantially to the global burden of disease, emphasizing the importance of periodontal assessment alongside dental caries [1]. Globally, most children have signs of gingivitis and, among adults, the initial stages of periodontal diseases are prevalent. Severe periodontitis, which may result in tooth loss, is found in 5%–15% of most adult populations. Aggressive periodontitis, a severe periodontal condition affecting individuals during puberty that leads to premature tooth loss, affects about 2% of youth [2].

Due to changes in demography and nutrition, developing nations like India have witnessed substantial alterations in the nutrition of children and adolescents throughout the last 20 years. Both nutritional deficiency and food excess have emerged as significant concerns in the double burden of malnutrition (DBM), which has been caused by factors like unequal food access, socioeconomic inequality, globalization, changing diets, and sedentary lifestyles [9, 10]. The increase in overweight and obesity creates more public health challenges. While malnutrition has traditionally been common in India, obesity is becoming increasingly prevalent. Childhood obesity is a major public health concern. According to the National Family Health Survey (NFHS), obesity in India has become an epidemic, impacting 5% of the population [3]. In addition, undernutrition is also a major concern, especially in rural populations [11–13].

BMI is a metric that compares body weight to height. It is commonly used to assess overweight or obesity levels [14]. Growth charts are essential for assessing children’s growth and nutrition. The WHO Growth Charts for children under 5 years and the Indian Academy of Pediatrics (IAP) 2015 Growth Charts for children aged 5–18 years are extensively used in India. Growth tracking from birth to age 18 is possible with a combined WHO–IAP height and weight chart [15]. A diet excessive in carbohydrates influences both dental caries and body mass index (BMI). Studies have linked dietary carbohydrate intake to obesity and refined carbohydrates to dental caries. However, connection between dental caries and obesity remains inconsistent. A systematic review of research from 1984 to 2004 found insufficient evidence to support a direct link between the two. Some studies suggest a link between BMI and dental caries [3, 7, 10, 11] while others do not [16, 17]. Even though this association has been studied, there is not enough data to draw definite conclusions. Willershausen et al. [18] discovered that students in elementary school had more caries lesions when their BMI was higher. However, Silva et al.’s [19] systematic review concluded that evidence linking obesity and dental caries was insufficient.

Although a number of Indian studies have demonstrated a link between dental caries and BMI, research specifically on their relationship is limited. With changing lifestyles, economic growth, reduced physical activity, and altered diets—especially in urban children—both dental caries and high BMI have become dominant public health concerns. It is crucial for medical practitioners to further explore the connection between caries and high BMI as sedentary lifestyles become more common. Understanding this relationship is essential for developing preventive strategies in schools, promoting healthy nutrition, and integrating oral health into broader child health policies. Therefore, the current study was conducted to assess the relationship between BMI and oral health, among children aged 12–15 years visiting a tertiary care hospital in Mangaluru, Karnataka. The study hypothesized that there was no association between BMI and oral health status among children aged 12–15 years.

## 2. Methodology

A descriptive, cross‐sectional study was conducted from September 2019 to July 2021, among 164 children aged 12–15 years attending the Department of Public Health Dentistry at a tertiary care hospital in Mangaluru, Karnataka. This study received ethical clearance from the Institutional Ethics Committee (Reference number YEC2/176).

The study participants were selected using consecutive sampling. All eligible children aged 12–15 years attending the outpatient department during the study period were included until the required sample size was achieved. Sample size was calculated based on a previous study [12], considering the following prevalence rates among adolescents—underweight (11.1%), normal weight (72.5%), and overweight (14.5%). With 90% study power and 5% significance level, it was found that 164 participants would be the minimum required sample size. The study included children between the ages of 12 and 15 years who consented to participate and whose parents provided written consent. Children not included were those undergoing orthodontic treatment, or if they had systemic diseases such as diabetes, asthma, epilepsy, thyroid disorders, cancer, tuberculosis, Addison’s disease, or Cushing’s syndrome as these conditions and their long term treatment protocol may independently influence BMI and oral health status, thereby acting as potential confounders [20–22].

### 2.1. Methods

Data was recorded using a structured proforma, which included the following details such as case number, date of examination, name, age, gender, socioeconomic status, address for communication, weight (in kilograms), and height (in meters).

### 2.2. Anthropometric Measurements

A uniform digital weighing equipment was used to determine the study participants’ body weight. A fractional weight value less than 500 g or more than 500 g was rounded to the closest whole number. A Stadiometer was used to measure the participants’ height, which was then recorded in meters. The formula used to calculate the BMI was 

BMI=Weight in kilogramHeight in meter square.

The BMI percentile for age and sex were plotted on the growth chart developed by IAP for 5–18‐year‐old Indian children [14]. Children were classified into four categories according to the revised IAP growth charts: underweight (≤3rd percentile), normal weight (>3rd percentile ≤23 adult equivalent), overweight (≥23 adult equivalent ≤27 adult equivalent), and obese (≥27 adult equivalent). Based on this classification, the study sample was categorized into three distinct groups namely Group I (underweight children), Group II (normal weight children), and Group III (overweight/obese children) as the number of obese children was small. The WHO Oral Health Assessment Form for Children, 2013 was used to assess oral status [23]. All assessments were performed by a single trained examiner using standardized WHO criteria, which minimized variability in measurements and reduced the risk of information and observer bias.

### 2.3. Oral Examination

Dental caries was recorded according to the dentition status criteria recommended in the WHO Basic Oral Health Survey Methods. Periodontal status was assessed using the modified Community Periodontal Index (CPI) criteria given in the WHO Oral Health Assessment Form for Children (2013). Presence or absence of gingival bleeding was recorded for all teeth by gently inserting the WHO CPI Probe into the gingival sulcus. Periodontal pocket scores and Loss of attachment were not recorded for the participants, in accordance with WHO recommendations [23]. No subgroup and sensitivity analyses were performed.

### 2.4. Statistical Analysis

Statistical Package for the Social Sciences Version 26 (IBM Corp., Armonk, N.Y., USA) was used to data analysis. For continuous variables, the mean and standard deviation were computed, and for categorical variables, the percentage and frequencies. The relationship between BMI and oral status measures was determined using the Chi‐Square test. One‐way ANOVA was performed to assess differences in mean values among the underweight, normal‐weight, and overweight categories. Pearson correlation test was done for finding the correlation between BMI and oral health status parameters. A *p*‐value of less than 0.05 was considered statistically significant.

## 3. Results

This study examined the relationship between oral health and BMI among 164 children aged 12–15 years visiting a tertiary care hospital in Mangaluru, Karnataka. A total of 170 children were approached for participation, of whom six did not provide consent, resulting in the final sample of 164 participants. Among them, 57.3% were boys and 42.7% were girls. By age, 30.5% were 12‐, 13.4% were 13‐, 22% were 14‐, and 34.1% were 15‐years of age. BMI assessment revealed that 17.7% were underweight, 44.5% had normal weight, and 37.8% were overweight/obese.

Table 1 shows overweight children had significantly higher averages for decayed teeth (DT), missing teeth (MT), filled teeth (FT), and the overall decayed, missing, and filled teeth (DMFT) score when compared to normal or underweight children. All differences are statistically significant with a *p*‐value of less than 0.01. There were no significant differences in dt scores across BMI groups, with an overall mean of 0.13 ± 0.33. However, ft scores showed a significant difference (*p* = 0.03), with normal weight group having the highest mean ft score (0.12 ± 0.33) as depicted in Table 2.

**Table 1:** Comparison of mean decayed teeth (DT), missing teeth (MT), filled teeth (FT), and DMFT scores in relation to BMI.

BMI	DT	MT (mean ± SD)	FT	DMFT
Under weight	2.83 ± 2.37	0.28 ± 0.45	0.28 ± 0.52	3.37 ± 2.54
Normal	2.29 ± 2.2	0.23 ± 0.56	0.14 ± 0.38	2.61 ± 2.34
Over weight/obese	4.79 ± 2.51	0.63 ± 0.77	0.68 ± 1.02	5.93 ± 2.85
*p*‐Value	<0.01 ^∗^	0.001 ^∗^	<0.01 ^∗^	<0.01 ^∗^

*Note:* Test applied: One‐way ANOVA.

^∗^ *p*‐value ≤0.05 statistically significant.

**Table 2:** Comparison of mean dt, ft, and dft scores in relation to BMI among the study population.

BMI	dt (mean ± SD)	ft (mean ± SD)	dft (mean ± SD)
Under weight	0.1 ± 0.31	0 ± 0	0.1 ± 0.31
Normal	0.12 ± 0.33	0.12 ± 0.33	0.25 ± 0.43
Over weight/obese	0.15 ± 0.35	0.03 ± 0.17	0.18 ± 0.38
*p*‐Value	0.84	0.03 ^∗^	0.23

*Note:* Test applied: One‐way ANOVA.

^∗^ *p*‐value ≤0.05 statistically significant.

Of the 164 participants, six (3.7%) had healthy gingiva, 16.5% had 1–5, 62.8% had 6–10, 13.4% had 11–15, and 3.7% had more than 15 bleeding sites. Bleeding site distribution varied significantly by BMI (*p*  < 0.001) (Table 3).

**Table 3:** Comparison of number of teeth with gingival bleeding sites among the study participants according to BMI.

BMI	Number of bleeding sites				
	0 *N* (%)	1–5 *N* (%)	6–10 *N* (%)	11–15 *N* (%)	>15 *N* (%)
Under weight	0 (0.0)	9 (5.5)	19 (11.6)	0	1 (0.6)
Normal	5 (3.0)	15 (9.1)	45 (27.4)	6 (3.7)	2 (1.2)
Over weight/obese	1 (0.6)	3 (1.8)	39 (23.8)	16 (9.8)	3 (1.8)
*p*‐value	0.001 ^∗^				

*Note:* Test applied: Pearson Chi Square test.

^∗^ *p*‐value ≤0.05 statistically significant.

**Figure 1:**
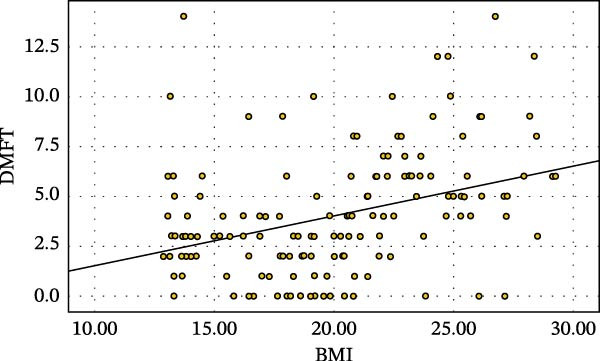
Correlation of DMFT with BMI among study participants.

A significant positive correlation (*r* = 0.329) was found between mean DMFT score and BMI (Figure 1). BMI also correlated positively with DT, MT, FT, and periodontal status, with coefficients of 0.234, 0.253, 0.377, and 0.34, respectively.

A small percentage of participants (3.7%), who were normal weight or overweight/obese, presented with dental erosion; there was no correlation with BMI (*p* = 0.266). Dental trauma was found among 25.6% of the study population; again, no correlation was detected between dental trauma among participants and their BMI.

Regarding urgency of intervention, prompt treatment was the most common requirement across all BMI groups (68.9%). Immediate (urgent) treatment due to pain or infection was most frequently required among overweight/obese children (9.8%), compared to normal‐weight group (3.7%) and underweight children (2.4%). Similarly, referral for comprehensive medical/dental evaluation was higher among overweight/obese participants (3.7%) compared to normal‐weight (0.6%) children, while no underweight child required a referral. Preventive/routine treatment needs were relatively higher among normal‐weight children (9.8%) compared to underweight and overweight/obese groups (0.6% each). The association between BMI and urgency of intervention was statistically significant (*p*  < 0.01).

## 4. Discussion

The goal of the current study was to assess the relationship between children’s oral health and BMI. In this study, participants were assigned into underweight, normal, or overweight groups based on their BMI, utilizing Growth Reference Charts provided by the IAP for Indian children. Although researchers commonly use the WHO Growth Reference Charts for individuals aged 5–19 years, these charts are unlikely to be suitable for the Indian cohort due to differences in child development patterns influenced by factors such as age at puberty, diet, environment, and genetics [24].

This study included 164 participants, with males comprising more than half of the group. This aligns with a study by Sharma and Kaur [25], which had 196 participants and a higher number of males than females. Additionally, the age range of participants in the current study, 12–15 years, was comparable to studies by Reddy et al. [10] and Goyal et al. [12], which involved participants aged 8–13 years and 12–18 years, respectively. In the present study, ~37.8% of the participants were overweight/obese, which is similar to the findings of Goodarzi et al. [7] where about 38.2% of participants were classified as overweight.

Dental caries is a common health issue, which serves as a significant risk factor for obesity in adulthood [26]. Severely underweight children with dental caries often experience rapid weight gain, and improved living standards after treatment [27]. Obesity and dental caries are complex conditions influenced by multiple factors, including diet, nutrients, oral hygiene, genetics, and lifestyle. While behavioral factors like excessive caloric intake and physical inactivity contribute to obesity, the relationship between obesity and dental caries is likely mediated by shared risk factors rather than a direct causal link [28]. According to a systematic review of research conducted between 2004 and 2011, half of the studies revealed no correlation between BMI and dental caries, one third found a positive association, and the remaining studies found an inverse relationship. These discrepancies could be caused by variations in study designs, study locations, methods of assessing nutritional status and dental caries, participants’ age, socioeconomic background, and other demographic characteristics [24]. Hayden et al. [29] noted that high BMI is associated with a higher prevalence of dental caries in developed but not in developing countries.

In the current study, a significant correlation was noticed between dental caries and BMI among participants. However, several studies have reported findings that differ from the present study. Swaminathan et al. [28] found no significant correlation between BMI and dental caries in children aged 3–12 years, mostly in the primary dentition stage, whereas our study focused on participants aged 12–15 years with predominantly permanent dentition. Differences in age, dentition stage, dietary patterns, cultural factors, and oral hygiene practices may explain this discrepancy, highlighting that caries risk is multifactorial and not solely dependent on BMI. Similarly, Sadeghi et al. [30] reported no significant association between BMI and dental caries among 12–15‐year‐old adolescents in Iran. Factors such as diet, physical activity, screen time, and fluoride exposure may have influenced these results, in addition to geographical and sample size differences. Sharma and Kaur [25], in contrast, observed higher caries in overweight and obese children, possibly due to differences in age, dentition, and sample characteristics. Freitas et al. [31] also found no association between overweight/obesity and dental caries, as normal‐weight adolescents showed higher caries activity, suggesting that dietary habits, oral hygiene, and lifestyle factors may influence the BMI–caries relationship. On the other hand, Liang et al. [16] reported a positive association between BMI and dental caries, comparable to our findings [19], and a meta‐analysis by Hayden et al. [29] also supported a strong link between BMI and caries in permanent dentition [26]. Most participants in our study had DMFT scores between 1 and 5, similar to Alghamdi and Almahdy [32], and Prpić et al. reported that higher BMI correlated with poorer dental health, aligning with our results [15].

In addition to dental caries, a statistically significant association was observed between BMI and gingival bleeding, with overweight children showing more bleeding sites and a positive correlation between BMI and periodontal status (*p*  < 0.001; *r* = 0.34). This association may be attributed to the chronic low‐grade inflammatory state in obesity, where adipose‐derived pro‐inflammatory mediators enhance periodontal inflammation, along with metabolic disturbances and suboptimal oral hygiene practices [33]. In contrast, Sapunarova et al. [34] reported no significant relationship between BMI and gingivitis, identifying oral hygiene and age as primary determinants; the discrepancy may be related to differences in age group and dentition stage, as their study involved younger children, whereas the present study focused on adolescents. Consistent with our findings, Sfasciotti et al. [35] observed poor periodontal health among overweight/obese children, while Sood et al. [36] and Assi et al. [37] found no significant association, possibly due to variations in study design, population characteristics, and periodontal assessment methods. This study found no correlation between dental erosion and BMI among participants, which contrasts with Tong et al. [38], in which obese children had a greater likelihood of dental erosion. This discrepancy can be attributed to Tong et al.’s [38] participants being recruited from obesity clinics, where consumption of sweetened carbonated drinks and increased intake of citrus fruits and juices for weight management could have contributed to dental erosion. In this study, 25.6% of participants experienced dental trauma, with no link found between trauma and BMI, consistent with Soriano et al. [39]. However, Basha et al. [40] reported an association with overweight, possibly due to their larger sample size. Similar to Bajjali and Rajab [41], obesity did not have an effect on occurrence of dental injuries.

Limitations of the study include a relatively small sample size, hospital‐centered design and consecutive sampling, restricting the generalizability of the results. Data regarding dietary behaviors, especially sugar intake, and oral hygiene behavior were not assessed, which may have influenced the observed relationship between BMI and oral health outcomes. The overweight and obese categories were combined due to the small number of obese participants, which may have masked potential differences between these two groups. The strengths of this study include strengths of the study included the use of standardized WHO oral health evaluation criteria and a complete dataset with no missing information.

Our study recommends further research with larger, multicenter samples to investigate dietary patterns, fluoride use, and oral health service utilization. Since obesity and oral health issues share modifiable factors like diet and lifestyle, regular dental check‐ups and education are essential for obese individuals. Promoting healthy nutrition and physical activity, alongside integrating weight counseling into dental practice, is crucial for preventing both obesity and oral diseases, to improve public health outcomes. For long‐term effects, it is advised to update dental curricula to highlight these associations, strengthen public health policies, and improve access to affordable preventive dental care.

### 4.1. Implications for Policy and Prevention

In order to identify children at risk for obesity‐related oral disorders, the results emphasize the necessity of integrated health policies that include routine BMI evaluation in school and dental health programs. Shared risk factors can be addressed by preventative policy initiatives such limiting sugar‐rich foods and drinks in school settings, encouraging physical exercise and a balanced diet, and expanding access to preventive dental care. Children’s long‐term oral and general health outcomes may be enhanced by incorporating nutrition and weight counseling into normal dental care.

## 5. Conclusion

The findings of this study indicate an association between BMI and dental caries as well as gingival status among children aged 12–15 years. These findings highlight the need for integrated preventive strategies that combine nutritional monitoring and oral health promotion in school and healthcare settings. Routine BMI and oral health assessments in pediatric care may support early identification and comprehensive risk reduction.

## Funding

This study was self‐funded by the authors. The article processing charge (APC) for publication was supported by Yenepoya (Deemed to be University).

## Disclosure

All scientific content, data analysis, interpretation, and conclusions are the original work of the authors. The authors take full responsibility for the accuracy and integrity of the manuscript.

## Conflicts of Interest

The authors declare no conflicts of interest.

## Data Availability

The datasets generated and analyzed during the current study are not publicly available to protect participant confidentiality. However, anonymized data can be obtained from the corresponding author upon reasonable request.
